# Coinfections identified from metagenomic analysis of cervical lymph nodes from tularemia patients

**DOI:** 10.1186/s12879-018-3218-2

**Published:** 2018-07-11

**Authors:** D. N. Birdsell, Y. Özsürekci, A. Rawat, A. E. Aycan, C. L. Mitchell, J. W. Sahl, A. Johansson, R. E. Colman, J. M. Schupp, M. Ceyhan, P. S. Keim, D. M. Wagner

**Affiliations:** 1 Pathogen and Microbiome Institute, Northern Arizona University, Flagstaff, AZ USA; 2 Department of Pediatric Infectious Disease Unit in Ankara, Hacettepe University Faculty of Medicine, Ankara, Turkey; 3 Translational Genomics Research Institute, Flagstaff, AZ USA; 4 Present Address: Division of Biomedical Informatics Research, Sidra Medical & Research Center, Doha, Qatar; 5 Present Address: Department of Epidemiology, Gillings School of Global Public Health, University of North Carolina, Chapel Hill, NC USA; 6 Department of Clinical Microbiology and Laboratory for Molecular Infection Medicine Sweden, Umeå University, Umeå, Sweden; 7 Present address: Department of Medicine, University of California, San Diego, California USA

**Keywords:** Coinfections, Concurrent infections, Tularemia, *Francisella tularensis*, Metagenomics, Fine-needle lymph node aspirate

## Abstract

**Background:**

Underlying coinfections may complicate infectious disease states but commonly go unnoticed because an a priori clinical suspicion is usually required so they can be detected via targeted diagnostic tools. Shotgun metagenomics is a broad diagnostic tool that can be useful for identifying multiple microbes simultaneously especially if coupled with lymph node aspirates, a clinical matrix known to house disparate pathogens. The objective of this study was to analyze the utility of this unconventional diagnostic approach (shotgun metagenomics) using clinical samples from human tularemia cases as a test model. Tularemia, caused by the bacterium *Francisella tularensis*, is an emerging infectious disease in Turkey. This disease commonly manifests as swelling of the lymph nodes nearest to the entry of infection. Because swollen cervical nodes are observed from many different types of human infections we used these clinical sample types to analyze the utility of shotgun metagenomics.

**Methods:**

We conducted an unbiased molecular survey using shotgun metagenomics sequencing of DNA extracts from fine-needle aspirates of neck lymph nodes from eight tularemia patients who displayed protracted symptoms. The resulting metagenomics data were searched for microbial sequences (bacterial and viral).

**Results:**

*F. tularensis* sequences were detected in all samples. In addition, we detected DNA of other known pathogens in three patients. Both Hepatitis B virus (HBV) and Human Parvovirus B-19 were detected in one individual and Human Parvovirus B-19 alone was detected in two other individuals. Subsequent PCR coupled with Sanger sequencing verified the metagenomics results. The HBV status was independently confirmed via serological diagnostics, despite evading notice during the initial assessment.

**Conclusion:**

Our data highlight that shotgun metagenomics of fine-needle lymph node aspirates is a promising clinical diagnostic strategy to identify coinfections. Given the feasibility of the diagnostic approach demonstrated here, further steps to promote integration of this type of diagnostic capability into mainstream clinical practice are warranted.

**Electronic supplementary material:**

The online version of this article (10.1186/s12879-018-3218-2) contains supplementary material, which is available to authorized users.

## Background

Underlying coinfections in primary infectious disease are an important variable to consider but are commonly undetected. A growing body of literature points to the high occurrence (10–50%) of coinfections [1–5], and > 75% of coinfections from diverse origins have an overall negative impact on human health [6]. Underlying coinfections complicate disease presentation [7, 8] and the ability to detect their presence is highly relevant to inform medical treatment. The under-diagnosis of coinfections is due, among other things, to a lack of clinical suspicion, overlapping symptoms, and/or the fact that traditional tools have limited ability to identify coinfections in the absence of a priori knowledge. Thus, exploration of new diagnostic strategies is necessary to advance the understanding of the contribution of coinfections to infectious disease manifestations and treatment responses.

Significant advances in next generation sequencing have recently made metagenomics, an unbiased shotgun method of analysis, a widely used tool in just about all areas in biology, including infectious disease diagnostics [9, 10]. Metagenomics is powerful for its ability to diagnose unsuspected microbial agents [11]. It directly analyzes samples in their entirety, which removes the requirement for a priori knowledge to obtain comprehensive information. In this capacity, metagenomics surpasses the limits encountered with traditional diagnostics.

Many infectious disease-causing microbes are considered foreign by the host immune system and, therefore, are actively routed to the lymph nodes. As a consequence, lymph nodes capture and house disparate microbes [12] regardless of their transmission route or ability to persist within the host. In a non-disease state, cervial lymph nodes are normally microbe-free environments [12]. Because of these unique attributes, lymph nodes make an ideal clinical target to detect underlying coinfections.

*F. tularensis* is the causative agent of the zoonotic disease tularemia and it can infect humans by several routes, including the ingestion of contaminated water or food. Exposure to *F. tularensis*-contaminated water [13, 14], blood-feeding vectors [15], or, on rare occasion, aerosolized particulates [16] each lead to distinct clinical forms of tularemia: orophyryngeal, ulcergrandular, and respiratory tularemia, respectively. In the rapidly developing nation of Turkey, tularemia has been on the rise since 2009 and oropharyngeal disease is the most common form [17]. This form involves a sore throat and the swelling of cervical lymph nodes. Antibiotic treatment is highly effective at significantly shortening disease duration [17] and very little evidence exists to support the idea that antibiotic resistant *F. tularensis* strains are prevalent in Turkey [18]. Because swollen cervical nodes are observed from many different types of human infections occurring in Turkey [19], we wanted to use cervical fine-needle aspirates of lymph nodes from eight tularemia patients [20] as a test model to analyze the utility of shotgun metagenomics to assess for the presence of multiple infectious agents.

## Methods

### Direct whole-genome sequencing of clinical lymph node samples

DNA extracts from fine-needle aspirates of lymph nodes from eight de-identified tularemia patients in Turkey [20] were processed in the Pediatric Infectious Disease Unit of the Faculty of Medicine, Hacettepe University hospital in Ankara, Turkey and subjected to direct metagenomics sequence analysis. The human fine-needle aspirates were collected as part of the medical workup for tularemia diagnosis and, therefore, were not subject to Institutional Review Board regulations; the residual aspirate materials were de-identified and donated to this study. The patient samples were selected based on sufficient levels of *F. tularensis* DNA as confirmed by PCR; *F. tularensis* isolates were not obtained from these eight patients. To prepare the libraries for metagenomics sequencing, 100 μL of DNA extract per clinical sample was processed using the KAPA Library Preparation Kits with Standard PCR Library Amplification/Illumina series (KAPA biosystems, Boston MA, code KK8201) with modifications (Additional file 1 - Methods); this kit is designed to target double stranded DNA and, therefore, RNA sequences were likely not captured in our study.

### Bioinformatic analyses

WGS data from the eight samples were analyzed using the metagenomics data analysis method MetageniE (https://github.com/ngsclinical/metagenie), as previously described [21] and with the following specific settings. We utilized quality filtration (PHRED quality score > 15, minimum length > 50, low complexity (dust) and removal of duplicates) with Prinseq [22]. The human filtration module processed reads with BWA [23] against a human reference genome (Hg19) to remove human reads, and the pathogen detection module utilized global aligner BWA and local aligner BLAT [24] on the filtered reads against bacterial and viral databases (Build 56 downloaded from ftp://ftp.ncbi.nih.gov/refseq/release/). Genome coverage of the mapped reads was visualized with Tablet [25]. The results were further confirmed with the metagenomic pipeline SURPI [26]. Paired end raw reads were concatenated and processed [26] with SURPI in “fast” mode with a d_NT_alignment value of 6. Read counts were tabulated from the SNAP [27] alignment against their custom reference genome database. Bioinformatics data were curated for the presence of bacteria and viruses. All raw reads were submitted to NCBI as Sequence Read Archives (SRA) (Table 1). To assess for inadvertent contamination from the environment of the sequencing facility, we bioinformatically analyzed other complex clinical and environmental samples processed at the same (Translational Genomics Research Institute; TGen) facility as the Turkish clinical samples. These samples were prepared and subsequently sequenced at the same time as the Turkish clinical samples utilizing the same reagents.

**Table 1:** *F. tularensis* positive clinical samples

NAU ID	Patient ID	WGS Bioinformatic sequence (read counts)						PCR status		
		NCBI accession #	*R. picketti*	*P. acnes*	*F. tularensis*	HBV	Parvovirus B-19	*F. tularensis*	HBV	Parvovirus B-19
F0739	3	SRR1925378	10	119	1960	30	2	+	+	+
F0742	6	SRR1925905	371	14	3265	0	6	+	–	+
F0741	5	SRR1925811	157	2	131	0	2	+	–	–
F0737	1	SRR1924572	89	1	260	0	0	+	–	–
F0738	2	SRR1925227	167	8	474	0	0	+	–	–
F0744	8	SRR1927285	3	7	1060	0	0	+	–	–
F0745	9	SRR1928041	38	0	835	0	0	+	–	–
F0749	13	SRR1931205	106	2	950	0	0	+	–	–

### Molecular confirmation of pathogens detected by bioinformatics analysis

To test for the presence of low level hepatitis B (HBV) and human parvovirus B19 (B19) in all eight clinical samples, we employed a nested PCR approach using assays developed using information from previous publications [28, 29] (Table 2), and confirmed the pathogen detection by Sanger sequencing of the final PCR amplicons. Nested PCRs for HBV and B19 were accomplished by two PCR amplification steps that employed the use of external primers followed by amplification with internal primers.

**Table 2:** Primer Sequence for nested PCR amplification

Pathogen Target	Nested PCR scheme	Primer Sequence	Amplicon size	Sanger Sequence target	Gene Target	Published
Hepatitis B_F1	Outer Forward	GGGAGGAGATTAGGTTAA	216 bp	NA	DistalX/pre-C gene	Chakravarty et al., 2002
Hepatitis B_R1	Outer Reverse	GGCAAAAAAGAGAGTAACTC				
Hepatitis B_F1	Internal Forward	*agctttccttgtttcgaattttataaTCTGTTCACCAGCACCAT	74 bp	37 bases		
Hepatitis B_R1	Internal Reverse	AGGCTTGAACAGTAGGACA				
HpB19_F1	Outer Forward	CAAAAGCATGTGGAGTGAGG	398 bp	NA	VP1	Koch and Adler et al., 1990
HpB19_R1	Outer Reverse	CTACTAACATGCATAGGCGC				
HpB19_F1	Internal Forward	CCCAGAGCACCATTATAAGG	288 bp	251 bases		Yamakawa et al., 1995
HpB19_R1	Internal Reverse	GTGCTGTCAGTAACCTG				

Amplification of the PCR product by the external primers was achieved in 10 μL reaction volumes using real-time PCR with the following conditions: 1 μL DNA extract, 2× SYBR green master mix (Life Technologies, Grand Island, NY) diluted with molecular grade water to bring final concentration to 1×, and 0.2 uM primers (Integrated DNA Technologies, San Diego, CA). A real-time PCR 7900 instrument (Life Technologies, Grand Island, NY) was programmed with the following protocol: 95 °C for 10 min to release the polymerase antibody, followed by 40 cycles of 95 °C for 15 s and 55 °C for 60 s. The PCR products from the external primers were diluted to 1:1000 prior to being used as a template for the next amplification step involving internal primers. Amplification of PCR with the internal primers (Table 2) was achieve in 10 μL reactions using conventional PCR with the following conditions: 1 μL of diluted PCR product (1:1000) as template, 1× PCR buffer, 2.5 mM MgCl_2_, 0.2 mM dNTPs, 0.16 U/ μL Platinum® *Taq* polymerase (Invitrogen, Carlsbad, CA, USA), and 0.2 μM of each primer. The thermocycle protocol was as follows: 94 °C for 10 min to release the polymerase antibody, followed by 35 cycles of 94 °C for 30 s, 55 °C for 30 s, and 72 °C for 60 s.

To confirm the true positive detection of HBV and B19 DNA from the clinical samples by internal primers of the nested approach, we generated Sanger sequences of the PCR amplicons. Sequencing was performed directly on the parvovirus B19 PCR product (251 bp) generated from the final B-19 internal primers (Table 2). The internal primers for HBV PCR generated a short amplicon (only 74 bp) within which only 37 bp represented the original HBV sequence present as the starting template in the clinical sample. Due to the exceptionally short HBV fragment size, we used a novel molecular strategy that incorporated this small PCR product into a larger fragment resulting in a 356 bp fragment (Additional file 1: Methods Figure S1), which was directly sequenced. The final HBV and B19 PCR products were treated with ExoSAP-IT (Affymetrix, Santa Clara, CA, USA) using 1 μL of ExoSAP-IT per 5 μL of PCR product under the following conditions: 37 °C for 15 min, followed by 80 °C for 15 min. Treated products were then diluted in the range of 1:2 to 1:5 depending on amplicon intensity (as determined by agarose gel electrophoresis). HPV-B19 was sequenced in both directions using BigDye® Terminator v3.1 Ready Reaction Mix (Life Technologies Applied Biosystems, Foster City, CA, USA) with the same forward and reverse primers from the initial PCR. HBV was sequenced in one direction with a forward primer (Elong-fwd356, ATATATTGTAACTAAACTATGTGCCGCTGA) that targeted the elongated region (Additional file 1: Methods Figure S1). We used 10 μL volumes for sequencing reactions containing the following reagents (given in final volumes): 3 μL of 5× Sequencing Buffer, 1 μL BigDye® Terminator v3.1 Ready Reaction Mix, 1 μL of a 10 μM primer stock, and 5 μL diluted PCR product. The following thermocycling conditions were used: 96 °C for 20 s, followed by 30 cycles of 96 °C for 10 s, 50 °C for 5 s, and 60 °C for 4 min. An ethanol precipitation technique was used to clean and precipitate the DNA pellet, and Sanger sequencing was carried out using an AB 3130xl® automated genetic analyzer (Life Technologies, Grand Island, NY); sequence chromatograms were edited manually in Sequencher 5.0 (Gene Codes, Ann Arbor, MI). Sequences were blasted in NCBI to search for perfect sequence matches with published Hepatitis B and Human Parvovirus B19 data.

As a positive control for our molecular approach, we constructed a synthetic sequence of 614 bp (Integrated DNA Technologies, San Diego, CA) encoding known HBV and B19 sequence regions targeted by the published assays [28, 29]. To confidently differentiate real signal from false signal due to potential cross contamination with our synthetic positive control, we engineered six deliberate point mutations not observed in nature within the PCR assay targets of the synthetic positive control (Additional file 1: Methods Figure S2). With this design, we were able to discern true positives from false positives after sequencing was performed based on the presence of the deliberate mutations. Water was added in place of template as negative controls, and all sample reactions were conducted in replicates of two.

## Results

**Fig. 1:**
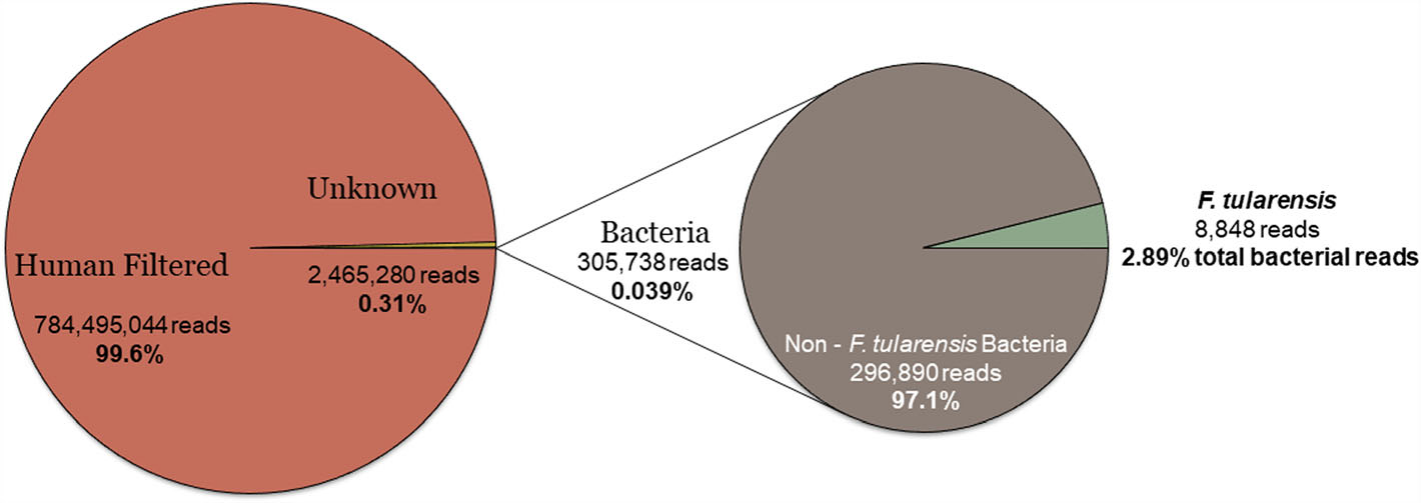
Direct sequence of eight clinical lymph node aspirate samples. Pie chart indicate 787,568,687 total reads compiled across the eight samples, providing percent partition of DNA sequences originating from human, unknown, unspecified bacterial, and Francisella tularensis. Read length were 100 bp at 1000× coverage depth

Metagenomics analysis of fine-needle aspirates of cervical lymph nodes from tularemia patients identified underlying coinfections (HBV and parvovirus B19). The true burden of coinfection may have been underestimated by not accounting for RNA viruses. Metagenomic analysis identified the presence of *F. tularensis* in all eight clinical samples when analyzed by both MetaGeniE and SUPRI. In addition, both analysis search methods detected other microbes in the same subset of patient samples (Parvovirus B19 in patients 3, 5, and 6, and HBV positive in patient 3, see Table 1). When combining total sequencing reads from all eight clinical samples, we obtained a total of 787,568,687 reads with 99.6% (784,495,044) matching human DNA, 0.31% unknown (2,465,280), and 0.039% (305,738) matching bacteria (Fig. 1). Among 305,738 reads from bacteria, 8848 reads matched *F. tularensis*, which comprised 2.89% of total bacterial reads (Fig. 1). This composition profile of extremely high levels of human DNA and low-level *F. tularensis* DNA in these clinical samples is consistent with our real-time PCR data (data not shown). Despite this extreme disproportionate ratio between human vs pathogen DNA species, 1000× sequence coverage provided enough sequences of *F. tularensis*, HBV, and parvovirus at high sequence match identity to solidly confirm the presence of these pathogens in specific clinical samples (Table 1). The other detected non-*Francisella* bacterial reads were classified as errors due to poor sequencing match identities with reference bacteria in published databases.

Our metagenomics analysis also detected non-pathogenic commensal skin bacteria, *Propionibacterium acnes*, which is likely real (Table 1) and is probably an incidential consequence of the fine-needle aspiration process itself, which involves the direct puncture of the skin [30]. We also detected *Ralstonia picketti* sequence in all eight patient samples (Table 1). Many clinical reagents, including ultra-pure water systems, have been reported to be contaminated with *Ralstonia* species [31, 32] and we hypothesize that this a likely source for our samples. Since *R. picketti* and *P. acnes* are known to have little clinical consequence [33, 34], no molecular confirmation was conducted on these organisms.

Our metagenomics analysis yielded high quality matches for 8848 sequencing reads of *F. tularensis* generated from all eight samples, which represents a very small fraction of the *F. tularensis* genome (see Table 1, Additional file 1: Methods Figure S3). Despite limited data for this analysis, more data could have been captured by this metagenomics strategy by increasing the coverage depth for which the sequencing was generated (i.e. > 1000× coverage). We found no evidence for *F. tularensis*, HBV, human parvovirus B19, and *Ralstonia* species among other clinical samples prepared and sequenced at the TGen sequencing facility, suggesting that the DNA sequence of these four microbes did not originate from the environment nor commercial reagents used in this facility at the time lymph node aspirates were processed.

Standardized traditional diagnostics independently confirmed the HBV coinfection in patient 3 that was initially detected through metagenomics. Active infection with HBV was confirmed in patient 3 via a serological diagnostic test, despite being missed by prior clinical examination. This confirmation was communicated using a method that retained the integrity of the patient de-identification system. No further information in respect to the stage of disease for this patient was obtained.

Molecular methods confirmed the presence of DNA sequence from multiple pathogens in three of the eight clinical samples, consistent with coinfection in these patients. We tested all eight samples that were PCR-positive for *F. tularensis* for the presence of parvovirus B19 and HBV. Through a combination of nested PCR followed by Sanger sequencing using parvovirus B19-specific primers [29], we confirmed detection from patients 3 and 6 but not 5 (Table 1). The 251 bp B19-specific amplicon from patients 3 had 100% sequence identity with published strains of human parvovirus B19 encoding a VP1 gene (EU478584), and the B19-specific amplicon from patient 6 had 99% sequence identity to published strains. This comparison identified a single base mutation that did not match any of the six deliberate mutations engineered in the synthetic positive control. Thus, this mutation either reflects the sequence of the original template or arose as an artifact introduced during PCR and sequencing process. An HBV-specific amplicon was generated from patient 3 and not from the other seven samples (Table 1). HBV-specific primers amplified a 37 bp fragment in patient 3 that perfectly matched published strains for C12 X protein (X) and core protein (C) genes (KP309751).

Not all pathogens initially detected by bioinformatics were confirmed through molecular methods. Parvovirus B19 was detected in patient 5 by metagenomics but not by our nested PCR Sanger sequencing molecular techniques. Although this suggests that the PCR assay used in our study is less sensitive than deep sequencing technology, it is thought that deep sequence Illumina output is nearly comparable to well optimized real-time PCR assay [35]. Thus, the sensitivity difference more likely stems from the technical differences between the two detection strategies. Unlike real-time PCR, which used 1 μL of DNA extract per reaction, metagenomics sequencing captured information from 100 μL of DNA extract. The results suggest that the larger volume of template enabled the capture of enough low-level parvovirus DNA in patient 5 for successful sequencing that was missed using the PCR strategy.

## Discussion

The importance of identifying underlying coinfection(s) is gaining greater appreciation [5, 6] but obtaining such information still remains challenging. We demonstrate an effective strategy to capture existing coinfections by using fine-needle aspirates obtained from cervical lymph nodes from tularemia patients. Other clinical sample types may be inferior at detecting coinfections as suggested by our finding that *F. tularensis* was PCR negative in blood samples of all eight patients (data not shown) but positive from the lymph node aspirates [20]. Using the metagenomics approach, we were able to detect diverse organisms (bacterial and viruses) that greatly differed in transmission routes and host persistence, indicating a lack of bias based on these differing biological parameters.

Surveying for pathogens from a clean microbial environment, such as lymph nodes, may be a good approach to diagnose clinically relevant microbes. However, not all diagnoses necessarily reflect active disease or an infection that has clinical relevance. There are pathogens, including parvovirus B19 DNA, that are never cleared but, rather, continue to persist in a dormant state in the host [36–38]. Studies have documented that parvovirus B19 DNA sequence is detectable, albeit at very low levels, from a wide range of human clinical samples (skin, synovium, tonsil, heart or liver [36] and bone marrow [39]) years after seroconversion. Although such surveys have not been conducted in lymph nodes, fine-needle lymph node aspirates contain cellular material, including immune cells continuously migrating between these nodes, the circulation system, and the bone marrow [40]. For this reason, we cannot conclude that the parvovirus B19 detected in three pediatric patient samples in this study was the result of acute infections. Initial patient medical examinations did not note signs of active skin rashes (data not shown). However, missed symptoms could be explained by the examination occurring during the early or late phase of this acute disease. In short, metagenomic diagnostics is highly informative for detecting unsuspected pathogens, but clinicians must continue to apply judgement to determine if detected pathogens have clinical relevance and/or warrant treatment.

Although fine-needle aspirates of lymph nodes are highly informative clinical samples, their availability varies. In Turkey, fine needle aspiration is considered routine as part of diagnosis and treatment for oropharyngeal tularemia and other diseases affecting lymph nodes [17, 41]. However, due to the clinical invasiveness of lymph node aspiration, in other countries this approach may be reserved for only those patient cases with lymphadenopathy of uncertain etiology.

Coinfections are not commonly considered when diagnosing and treating tularemia and, therefore, the clinical significance of coinfections is uncertain. Our results, however, indicate that coinfections are not rare in tularemia patients in Turkey. In fact, the rates of HBV and human parvovirus in our pediatric tularemia patients coincide well within the overall prevalence rates of these two diseases in the general Turkish population (10 and 21%, respectively) providing some indirect evidence that the detection could be unrelated to the acute *F. tularensis* infection [42, 43]. Very little is known regarding the effects of coinfection on clinical manifestation of tularemia and it is beyond the scope of this study to glean insight as to the clinical significance of tularemia patients with the identified coinfections.

## Conclusions

Our study reveals that shotgun metagenomics targeting fine-needle lymph node aspirate samples is a promising clinical diagnostic strategy to identify underlying coinfection in primary disease as demonstrated by our ability to simultaneously detect *F. tularensis* and possible coinfections. Other clinical specimens such as blood may not be as informative for this purpose. In-depth exploration of new broad diagnostic methods that identify multiple microbes and possible coinfections is an important first step to advance the understanding of disease manifestations and treatment responses, and to possibly promote this capability into mainstream clinical practice.

## Additional file

Additional file 1: Methods. Whole genome sequencing of clinical lymph node samples. Molecular confirmation of pathogens detected by bioinformatics analysis. Metagenomic bioinformatics analysis. (DOCX 70 kb)

## Abbreviations

AZ TGen: Arizona Translational Genomics
HBV: Hepatitis B Virus
HPV: Human Parvovirus
HPV-B19: Human Parvovirus B19
NCBI: National Center for Biotechnology Information
PCR: Polymerase Chain Reaction
SRA: Sequence Read Archive
