# Supplementary material for: Coinfections identified from metagenomic analysis of cervical lymph nodes from tularemia patients
Source: BMC Infect Dis. 2018 Jul 11;18:319. doi: 10.1186/s12879-018-3218-2 (PMC6042416; doi:10.1186/s12879-018-3218-2)
Supplement: Supplementary file 1 — Methods. Whole genome sequencing of clinical lymph node samples. Molecular confirmation of pathogens detected by bioinformatics analysis. Metagenomic bioinformatics analysis. (DOCX 70 kb) [file 12879_2018_3218_MOESM1_ESM.docx]

**Supplemental Materials: Methods**

***Whole genome sequencing of clinical lymph node samples****.* Libraries were prepared for sequencing on a HiSeq 2000 at TGen North facility using the following methods: 100 µL of DNA extract per clinical sample was processed using KAPA Library Preparation Kits with Standard PCR Library Amplification/Illumina series (KAPA biosystems, Boston MA, code KK8201) with the following modifications: the adapters and 8bp index oligos purchased from IDT® (Intergrated DNA technologies, Iowa) based on Kozarewa and Turner, 2011 (1), were used in place of those supplied in the Kapa preparation kit. Approximately one microgram of DNA per sample was fragmented using a SonicMan (Matrical) using the following parameters: 75.0s pre chill, 16 cycles, 10.0s sonication, 100% power, 75.0s lid chill, 10.0s plate chill, and 75.0s post chill. The fragmented DNA was purified using Qiagen Qiaquick PCR purification columns (Qiagen, cat. no. 28104) and eluted into 42.5 μL of Elution Buffer. The A-tailing step was conducted at 37**°**C for 30 mins. The adapter ligation used 1.5 μL of the 40 μM adapter oligo mix (1) only one post- ligation bead cleanup was completed. All purification steps were completed with the 1.8x SPRI bead protocol in the KAPA protocol. Size selection of fragments was gel based; 30 μL of clean ligated material was run in a 2% agarose gel. Several gel slices, corresponding to different average DNA fragment sizes (220 and 600 bp fragments), were extracted from the gel and purified with a Qiagen Gel Extraction kit (Qiagen, cat. no. 28704) and eluted in 30 μL of Elution Buffer. PCR was optimized to improve yield and genome coverage.  Two μL of DNA, two μL of 10 μM both primers, 25 μL of Kapa 2X HIFI PCR Master Mix (KAPA biosystems), and 19 μL of H2O were combined. The following PCR parameters were used: initial denaturation 2 min at 98°C, 12 cycles of 30 sec at 98°C, 20 sec at 65°C, 30 sec at 72°C, with a final extension of 5 mins at 72°C. The samples were sequenced using 2x100 bp run on the Illumina HiSeq 2000 instrument (1000x coverage). Each read was 100 bp in length from a 600 bp insert size and paired reads.

***Molecular confirmation of pathogens detected by bioinformatics analysis****.*

***Novel Fragment Elongation Strategy to perform Sanger Sequencing for short HBV fragment.*** Due to the exceptionally short HBV fragment size, we used a novel molecular strategy that incorporated this small PCR product into a larger fragment resulting in a 356 bp fragment. We stitched together two fragments into a single longer fragment through PCR strategy. We generated a 74 bp HBV amplicon product using the internal forward and reverse primers of the nested PCR scheme (Table 2). The forward primer was modified by the addition of 26 bp of non-HBV sequence at the 5’ end (lower case and green highlight) (agctttccttgtttcgaattttataaTCTGTTCACCAGCACCAT). This non-HBV sequence tail became incorporated into the final small HBV-specific amplicon and served to incorporate the HBV-specific amplicon into a larger fragment. The larger fragment (elongation gBlock), generated by IDT as a genomic block, contained the same non-HBV sequence (agctttccttgtttcgaattttataa) incorporated in the 74 bp HBV amplicon at the end of the fragment (Fig S1).

**Fig S1:** Schematic design of the Elongation gBlock. This gBlock contains sequence targets that permits incorporation of the 74 bp HBV-specific amplicon with the gBlock (highlighted in green). Yellow highlight represent the forward primer that enabled the amplification of the enlongated product.

CATAGTTACATGAGCTATCACAACAGTAGTTGTACCACGCTCGTGTGACCAGCCAATTAAAcGTTGTAAGGTTGAAAACATCAATAGTAACCTTTGCCTGAATCTCTATAtATTGCGATCAATGCATACATTGGTAATGATAATAACTCTAGACCAACATATATTGTAACTAAACTATGTGCCGCTGATAGTTTCGGTGATACCAACTACTaGTATATTATTTTTCTTAGCAACTTCCAAGACATCACTTGTAACATTATCTGTGACTTGTTTGGCTATAAAAATTATAACTATCATAATTTTTATAATAATAGGAATTGGTATTATTaGTGGTTTTTTACATTCTAATAAGCCTTCATTCAAAGACATTTATTATCTCCTcTTAAATATTTTCTAAACTATCAATACTGTTTGTATCTTTTATATCTATTGATTTATCTATAGCTTTCCTTGTTTCGAATTTTATAA

To increase the length of our sequence target, we stitched together the 74 bp HBV-specific amplicon with gBlock using a PCR strategy that utilized the common overlapping sequence (agctttccttgtttcgaattttataa) to incorporate the two fragments into a single fragment. This PCR included a forward primer (Elong-Fwd356) that targeted regions of the gBlock (Elong-fwd356,ATATATTGTAACTAAACTATGTGCCGCTGA). In this PCR, Elong-Fwd356 functioned as the forward primer, the 74 bp HBV specific amplicon functioned as the reverse primer, and the Elongation gBlock functioned as the template. The PCR was 20 μL total volume with final concentrations 1x PCR buffer, 2.5 mM MgCl_2_, 0.2 mM dNTPs, 0.12 U/ μL (2.4U/20 μL) Platinum® *Taq* polymerase (Invitrogen, Carlsbad, CA, USA), 1 μL elongation gBlock (1/1000^th^), 0.2 mM Elong-fwd356 primer, and 1 μL HBV amplicon. The thermocycle protocol was as follows: 95°C for 5 minutes to release the polymerase antibody, followed by 30 cycles of 95°C for 30 seconds, 55°C for 30 seconds, and 72°C for 60 seconds.

***Engineered positive control***. We used a single 614 bp fragment (Fig S3), generated by IDT, to encode target sequences for HBV and parvovirus B-19.

**Fig S2:** Schematic design of the gBlock displaying the sequence of the synthetic positive control. Yellow and green highlights represent forward and reverse primers for HBV and B-19 region, respectively. Underlined sequences indicate primer sequence targets for the inner primers (forward and reverse) for each pathogen target. Lower case red letters represent engineered mutations not found in published sequences to enable the differentiation of product generated from the positive control from true positives.

**HepB,HpB19_gBlock**, GGGAGGAGATTAGGTTAAAGGTCTTTGTACTAGGAGGCTGTAGGCATAAATTGGTCTGTTCACCAGCACCAT GCAACaTTaTCCCgTCTGgCTAtTCATgTCATGTTCATGTC

CTACTGTTCAAGCCTCCAAGCTGTGCCTTGGGTGGCTTTGGGGCATGGACATTGACCCGTATAAAGAATTTGGAGCTTCTGTGGAGTTACTCTCTTTTTTGCCCAAAAGCATGTGGAGTGAGGGGGCCACTTTTAGTGCCAACTCTGTGACTTGTACATTTTCTAGACAGTTTTTAATTCCATATGACCCAGAGCACCATTATAAGGTGTTTTCTCCCGCAGCAAGTAGCTGgCACAATGCCAGTGGAAAGGAGGCAAAGGTTTGCACgATTAGTCCCATAtTGGGATACTCAACCCgATGGAGATAaTTAGATTTTAATGCTTTAAACTTATTTTTTTCACCTTTAGAGTTTCAGCACTTAATTGAAAATTATGcAAGTATAGCTCCTGATGCTTTAACTGTAACCATATCAGAAATTGCTGTTAAGGATGTTACAGACAAAACTGGAGGGGGGGTGCAGGTTACTGACAGCACTACAGGGCGCCTATGCATGTTAGTAG

***Metagenomic bioinformatic analysis.***

Our metagenomics analysis yielded high quality matches for 8,848 sequencing reads of *F. tularensis* generated from all eight samples combined. This represents a very small fraction of *F. tularensis* genome (Fig S3).

**Fig S3:** Genomic coverage of *F. tularensis* reads obtained from the eight clinical samples when aligned on the LVS reference strain. Red lines represent *F. tularensis* sequences detected from the clinical samples that overlap with the genomic regions of the LVS reference strain.


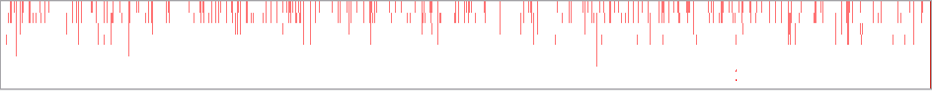


**Reference:**

1. **Kozarewa I, Turner DJ.** 2011. 96-plex molecular barcoding for the Illumina Genome Analyzer. Methods in molecular biology **733:**279-298.
